# Population Genomics of Chinese *Melia azedarach* Germplasm: Latitudinal Genetic Structure, Spatial–Climatic Associations, and Climate-Associated Candidate Genes

**DOI:** 10.3390/life16101582

**Published:** 2026-09-22

**Authors:** Yang Liu, Mengyi Chen, Wancun He, Haolin Zhang, Wei Zhao, Tingting Zou, Maoyin Shi, Juan Liu

**Affiliations:** 1 Jiangxi Provincial Key Laboratory of Subtropical Forest Resources Silviculture, Jiangxi Agricultural University, Nanchang 330045, China; 2 Jiangxi Forestry Technology Promotion and Education Center, Nanchang 330038, China

**Keywords:** *Melia azedarach*, population genomics, genetic diversity, clinal variation, spatial–climatic associations, genome-wide association study

## Abstract

To understand how geography shapes the genetic variation of *Melia azedarach*, we sequenced the whole genomes of 91 accessions from 91 maternal half-sib families across 15 provenances maintained in the National Germplasm Bank of China, an ex situ collection representing conserved germplasm. Genetic diversity increased from south to north (*He:* 0.098 to 0.333), whereas the Hainan population showed relatively low within-population diversity and, as confirmed by pairwise *Fst*, was highly differentiated from the mainland, forming an independent group. A Mantel test confirmed a significant isolation-by-distance (IBD) pattern (*r* = 0.307, *p* = 0.001). Redundancy analysis (RDA) explained 17.23% of the individual-level genetic variance, but a partial redundancy analysis (partial RDA) showed that most of this association was attributable to population structure, with the environmental variables explaining negligible independent variance after conditioning on population structure. Genome-wide association analysis (GWAS) further identified candidate loci potentially associated with climate-related traits, including a temperature-associated gene encoding a leucine-rich receptor-like protein kinase and a precipitation-associated gene encoding the stress-related protein RAR1. Together, these results map the genome-wide genetic diversity and spatial–climatic associations of *M. azedarach.* This study provides a genetic basis for evaluating banked resources, molecular breeding, and protecting this important tree species.

## 1. Introduction

*Melia azedarach* L. (*Meliaceae*) is a deciduous tree species widely distributed across tropical and subtropical regions. Native to East and Southeast Asia, it has been introduced and naturalized in many other parts of the world [1,2]. This species is fast-growing and widely used for timber production, urban landscaping, and ecological restoration [3,4]. Its wood is durable and resistant to decay, making it suitable for furniture, construction, and agricultural implements [5,6]. The species also has considerable medicinal and agrochemical value: compounds isolated from its bark and fruits, including limonoids such as toosendanin, have been used in traditional medicine and as natural botanical pesticides for biological pest control [7]. Given its economic and ecological importance, understanding the genetic diversity and population structure of *M. azedarach* is essential for the conservation, breeding, and utilization of its germplasm resources, particularly under ongoing climate change.

Many studies have assessed the genetic diversity of *M. azedarach* using morphological and molecular markers. For morphological traits, Cheng et al. [8] detected a northeast–southwest geographical gradient across 24 populations, which were divided into five major regions. Similarly, Chen et al, [9], and Liao et al. [10] observed north–south variation in seed and fruit traits across 17 provenances, suggesting that geographic isolation drives phenotypic differentiation. At the molecular level, studies based on simple sequence repeats (SSRs) have described similar patterns. Wan et al. [11] reported moderate genetic differentiation in Jiangxi populations, with diversity decreasing from southwest to northeast, and [12], using transcriptome-derived SSRs, showed that most genetic variation exists within rather than among provenances. However, these low-density markers provide limited genome coverage and cannot reliably identify loci under selection or separate the effects of genetic drift from adaptive selection.

Whole-genome resequencing (WGS) overcomes these limitations by providing millions of high-density single-nucleotide polymorphisms (SNPs) [13], which allow genome-wide variation to be characterized and evolutionary trajectories and population structure to be studied. Although population-scale WGS has been applied to study adaptation and gene function in woody species such as *Prunus cerasoides* [14], *Glycine soja* [15], and *Populus* spp. [16], it has not yet been applied to *M. azedarach*. Now that a chromosome-level reference genome is available for this species, WGS can provide a more complete basis for understanding its evolutionary history and spatial–climatic associations, which is the aim of the present study.

To protect and utilize forest genetic resources, China has established a network of National Germplasm Banks. Because the accessions preserved in these banks are maintained ex situ, their genetic structure reflects the conserved germplasm rather than that of contemporary wild populations, a distinction that should be kept in mind when interpreting molecular studies of banked material [17]. There has been extensive research on these ex situ resources, largely focused on basic phenotypic evaluations, including growth, fruit, and seed trait characterization across banked provenances [18,19]. Nevertheless, because all accessions are grown under uniform cultivation conditions, these ex situ repositories can function as common-garden platforms in which environmental noise from wild habitats is minimized, allowing evolutionary signatures driven by genetic inheritance to be studied more clearly than temporary environmental plasticity [20,21]. Moreover, the documented provenance origins and uniform cultivation conditions provide a valuable framework for detecting population genetic differentiation with minimized environmental confounding.

In this study, we investigated the *M. azedarach* germplasm preserved in the National Germplasm Bank (Jiangxi, China). Using population-level WGS, we analyzed the genome-wide variation of 91 accessions representing 15 different provinces. Our goals were to: (1) calculate genetic diversity and determine phylogenetic relationships; (2) describe population structure and test the effects of Isolation-by-Distance (IBD) and Isolation-by-Environment (IBE) models; (3) identify the climatic variables most strongly associated with genomic variation using redundancy analysis (RDA); and (4) conduct genome-wide association analysis (GWAS) to identify candidate loci associated with climate-related traits. This study provides a genetic basis for evaluating banked resources, molecular breeding, and protecting this tree species.

## 2. Materials and Methods

### 2.1. Experimental Materials

All experimental materials in this study were obtained from the National Germplasm Resource Bank of *M. azedarach* (115.62° E, 28.94° N), situated in Yongxiu, Jiangxi Province, China. Established in 2018, this repository systematically preserves germplasm collected from well-spaced mature plus-trees across the species’ natural distribution. The collection comprises open-pollinated maternal half-sib families, with multiple sibling individuals propagated and maintained per family line under uniform nursery and field conditions since 2021.

In the repository, each family line contains multiple sibling individuals. To prevent sampling bias caused by close familial co-ancestry (such as full- or half-sib relatedness) and to ensure that all sequenced accessions represent genetically independent operational units, our sampling strategy strictly selected only one healthy representative individual per maternal family. Consequently, a total of 91 accessions from 91 distinct families across 15 geographic provenances were included in this study. These provenances span a broad environmental gradient ranging from arid, cold northern margins to tropical southern islands, with the number of sampled accessions per provenance varying from 2 to 10 due to historical collection and propagation rates (Table 1). In October 2024, healthy young leaves from the current-year growth were sampled, immediately dried in silica gel, and preserved for subsequent genomic DNA extraction and whole-genome resequencing.

### 2.2. DNA Extraction and Whole-Genome Resequencing

Genomic DNA was extracted from the 91 leaf samples using the conventional CTAB method [22]. For each sample, sequencing libraries were prepared using the TruSeq™ Nano DNA Sample Prep Kit (Illumina, San Diego, CA, USA) following the manufacturer’s protocols. Briefly, 1 μg of DNA was fragmented to ~350 bp using a Covaris M220 ultrasonicator (Covaris, Inc., Woburn, MA, USA). After adapter ligation and PCR enrichment, libraries were sequenced on the Illumina NovaSeq 6000 platform (Illumina, Inc., San Diego, CA, USA). This yielded 2 × 150 bp paired-end reads with a target average depth of 10 × per individual and a quality filter of *Q*30 > 90%.

### 2.3. Sequencing Data Quality Control and Variant Detection

Raw reads were processed using fastp (v0.23.2) to remove adapters, low-quality bases (*Q* < 20), reads with >10% N content, and trimmed reads < 75 bp. Clean reads were aligned to the *M. azedarach* reference genome Melaze_AO_1.1 (NCBI Assembly ID: GCA_028052125.1) using BWA (v0.7.12-r1039) with the mem -k 32 parameter. This chromosome-level reference genome has a total length of 230.84 Mb anchored onto 14 pseudochromosomes, with a scaffold N50 of 16.92 Mb and a contig N50 of 3.13 Mb, providing a reliable framework for subsequent variant detection. Alignments were converted to BAM format, sorted, and merged using SAMtools (v1.14), and PCR duplicates were removed via Picard (v1.92). Sequencing depth and coverage statistics were calculated using custom Perl scripts. Average missing genotype rate and transition/transversion (Ti/Tv) ratio were calculated from the deduplicated BAM files and the filtered SNP set using Picard and VCFtools (v0.1.16), respectively. Variant calling was performed using GATK (v4.1.2.0) HaplotypeCaller. Raw variants were filtered via GATK VariantFiltration with standard criteria: QD < 2.0, FS > 60.0, MQ < 40.0, SOR > 3.0, MQRankSum < −12.5, and ReadPosRankSum <−8.0. VCFtools (v0.1.16) was then used to retain only biallelic SNPs with QUAL ≥ 20 and an individual depth (DP) ≥ 4. Finally, variants were annotated using ANNOVAR (v2019Oct24).

### 2.4. Data Processing and Population Genetics Analysis

Filtered SNPs were further pruned using VCFtools (v0.1.16) and PLINK (v1.90b) to retain only biallelic variants with a minor allele frequency (MAF) ≥ 0.05, a genotyping missing rate < 20%, QUAL ≥ 20, and an individual depth ≥ 4. We did not apply a strict LD-pruning step (e.g., PLINK --indep-pairwise with a window, step, and r^2^ threshold); all SNPs passing the above filters were used directly for the downstream analyses, because the NJ-tree, PCA, and ADMIXTURE analyses are individual-based and relatively insensitive to marker linkage, and retaining the full SNP set maximizes the genomic resolution of these analyses. Population genetic parameters, including expected heterozygosity (*He*), observed heterozygosity (*Ho*), polymorphism information content (*PIC*), nucleotide diversity (π), and the effective number of alleles (*Ne*), were calculated using VCFtools (v0.1.16) and PLINK (v1.90b) based on the distinct provenance groups. Among them, observed heterozygosity (*Ho*) is the proportion of heterozygous genotypes observed at a locus, averaged across all loci examined, whereas expected heterozygosity (*He*) is the probability that two alleles drawn at random from a population are different, representing the expected proportion of heterozygotes under Hardy-Weinberg equilibrium. Because the number of accessions varied among provenances (2–10 individuals), we applied rarefaction to standardize all populations to the minimum sample size (*n* = 2); the rarefied effective number of alleles (*Ne***^†^**) and expected heterozygosity (*He***^†^**) were then recalculated from the rarefied dataset to allow unbiased comparisons of genetic diversity among provenances, and these corrected values are presented together with the raw estimates in Table 2.

For genetic clustering, a neighbor-joining (NJ) phylogenetic tree was constructed using TreeBest (v1.9.2) based on the core SNP set under the *p*-distance model; the tree was unrooted and no outgroup was specified. Principal component analysis (PCA) was performed using GCTA (v1.93.2). Model-based ancestry clustering was inferred using ADMIXTURE (v1.3.0) with clusters (*K*) tested from 1 to 9. One independent run was performed for each *K* value (nine runs in total, one for each of *K* = 1 to 9), using the ADMIXTURE default convergence criterion (log-likelihood change < 1 × 10^−4^, with a maximum of 500 iterations) and a fixed random seed (seed = 42). The optimal *K* was selected as the value with the lowest cross-validation (CV) error, computed by the built-in 5-fold cross-validation.

Isolation-by-distance (IBD) was evaluated by correlating geographic distance (km) with the Euclidean genetic distance between accessions, computed from their genome-wide SNP genotypes, using Mantel tests (999 permutations) in the R package vegan (v2.6-4). IBD and isolation-by-environment (IBE) represent two alternative drivers of genetic differentiation: IBD predicts that genetic divergence increases with geographic distance because of limited gene flow, whereas IBE predicts that divergence is driven by environmental heterogeneity and divergent selection among populations in contrasting environments.

To test environmental association, a redundancy analysis (RDA) was performed in vegan using two spatial coordinates (longitude and latitude) and seven bioclimatic variables: bio1 (annual mean temperature), bio3 (isothermality), bio8 (mean temperature of the wettest quarter), bio13 (precipitation of the wettest month), bio14 (precipitation of the driest month), bio15 (precipitation seasonality), and bio18 (precipitation of the warmest quarter); bio1, bio3, and bio8 are temperature-related, whereas bio13, bio14, bio15, and bio18 are precipitation-related. All 19 BIOCLIM variables were initially extracted from the WorldClim database and screened for multicollinearity by Spearman correlation; variables with an absolute correlation coefficient > 0.8 were removed, retaining the more ecologically representative variable, as judged by its explanatory power in univariate RDA, the magnitude of its loading on the major RDA axes, and its ecological interpretability. The variance inflation factor was not used because the correlation-based approach is widely applied in environmental-association studies. The relative contribution of each variable was evaluated from the magnitude of its loading on the significant canonical axes and its ecological interpretability. The significance of the RDA models and axes was tested via ANOVA with 999 permutations.

Because the individual-level RDA may be influenced by the strong population structure, two complementary analyses were performed in vegan (v4.3.0): a partial RDA, in which the first five principal components of the genome-wide SNP data (cumulatively explaining 32.3% of the genetic variation) were included as conditioning variables, and a population-level RDA, in which individuals were pooled into the 15 provenances to form an allele-frequency matrix, used as a cross-validation of the individual-level result. Because accessions were pooled by province (only four unique environmental combinations), the bioclimatic variables are completely collinear with longitude and latitude and are therefore treated as aliased in RDA; consequently, only three non-collinear and ecologically representative variables (longitude, latitude, and annual mean temperature, bio1) were retained for model fitting. Candidate adaptive SNPs were defined as loci whose loadings on the first two RDA axes exceeded three standard deviations [23], and high-confidence candidates were taken as the intersection of the partial RDA and population-level RDA results.

### 2.5. Genome-Wide Association Analysis and Candidate Gene Identification

Two climate-related traits were used as target traits: annual mean temperature (bio1) and annual precipitation (bio13). The values for each accession were obtained from the WorldClim database according to the geographic coordinates of the 91 accessions. Phenotypic data were organized using Microsoft Excel, and the distribution of each trait was assessed in R by normality tests and frequency-distribution histograms.

Based on the whole-genome resequencing SNP dataset of *M. azedarach*, a genome-wide association study (GWAS) was performed using the rMVP package (version 1.4.6) in R. Three models were applied for each trait: the general linear model (GLM), the mixed linear model (MLM), and the fixed and random model circulating probability unification (FarmCPU), with a significance threshold of −log10(P) ≥ 6. Among the three models, FarmCPU detected the fewest significant SNPs and concentrated them at the most reliable associated loci, and was therefore selected as the primary basis for subsequent candidate gene identification. The results were visualized with Manhattan plots and quantile-quantile (QQ) plots generated via rMVP. To control for population structure and relatedness, principal components from PCA and a kinship matrix K (calculated using the VanRaden method) were included in the mixed models. Candidate genes were searched within a 50 kb window upstream and downstream of each significant SNP using the reference genome annotation (Melaze_AO_1.1, GCA_028052125.1), and their functions were annotated.

## 3. Results

### 3.1. Whole-Genome Resequencing and Population Genetic Variation Detection

Whole-genome resequencing of the 91 *M. azedarach* accessions yielded an average sequencing depth of 11.67× (SD = 1.41) across the entire population. On average, 93.98% of the reads were successfully mapped to the reference genome, with 88.48% of the genome covered at least once. The per-provenance missing genotype rate ranged from 5.24% (JH) to 17.77% (ZZ), and the transition/transversion (Ti/Tv) ratio ranged from 2.01 (AK) to 2.13 (SY), both within the ranges typically observed for high-quality whole-genome SNP data. Detailed per-provenance sequencing depth and quality-control statistics are provided in Table A1. Subpopulation average depths were 12.04× (SD = 1.09) for Hainan, 11.37× (SD = 0.52) for Hubei, 11.64× (SD = 0.69) for Jiangsu, and 11.54× (SD = 2.02) for Shaanxi. After filtering to exclude loci with a MAF < 0.05 or a missing rate > 20%, a core set of 6,695,869 high-confidence SNPs was retained for downstream analyses.

These identified SNPs were relatively uniformly distributed across the 14 reference pseudochromosomes (Figure 1). The total SNP count per chromosome correlated with chromosome length, with Chromosomes 1, 2, and 3 containing the highest densities and total variant numbers. Functional annotation showed that 39.26% of the SNPs were located in intergenic regions, followed by intronic regions at 35.24% (Figure 2). Upstream and downstream regulatory regions harbored 9.24% and 9.20% of the SNPs, respectively. Exonic regions contained 3.87% of the variants, while the remaining SNPs were distributed in the 3′ UTR (2.03%), splice sites (0.91%), and 5′ UTR (0.25%).

### 3.2. Population Genetic Diversity Analysis

Because the number of accessions varied among provenances (2–10 individuals), the raw diversity estimates of the two smallest provenances, Sanya (SY) and Chibi (CB), each represented by only two accessions, would be statistically unstable if compared directly with the larger populations. To address this small-sample problem and place all provenances on an equal footing, we applied rarefaction, standardizing every population to the minimum sample size (*n* = 2). The rarefaction-corrected effective number of alleles (*Ne***^†^**) and expected heterozygosity (*He***^†^**) were therefore calculated for all 15 provenances, including SY and CB, and are presented together with the raw estimates in Table 2.

In the raw estimates, genetic diversity varied clearly among provenances, with Shaanxi populations generally showing the highest values. The effective number of alleles *(Ne)* ranged from 1.1666 (SY) to 1.5825 (MX), and expected heterozygosity *(He)* ranged from 0.0974 (SY) to 0.3326 (AK), with the lowest values consistently found in southern and coastal populations. Observed heterozygosity *(Ho)*, defined as the proportion of heterozygous genotypes observed at a locus averaged across all loci examined, was highest in MX (0.3909) and lowest in JH (0.1299). Polymorphism information content (PIC) and nucleotide diversity (π) followed the same general trend, with π peaking in MX (0.3584) and being lowest in DF (0.1281).

After rarefaction, the corrected estimates (*Ne***^†^** and *He***^†^**) showed the same overall geographic pattern, confirming that the south-to-north increase in diversity was not an artifact of the uneven sampling. AK retained the highest rarefied diversity (*Ne***^†^** = 1.2357, *He***^†^** = 0.2400), followed by ZZ (*Ne***^†^** = 1.1923, *He***^†^** = 0.1982) and YX (*Ne***^†^** = 1.1793, *He***^†^** = 0.1850), whereas the lowest rarefied values were found in the southern and coastal populations, including NT (*Ne***^†^** = 1.0528, *He***^†^** = 0.0541), DF (*Ne***^†^** = 1.0568, *He***^†^** = 0.0579), CW, and LG. Notably, the high raw estimates of MX were reduced after rarefaction (*Ne***^†^** = 1.1653, *He***^†^** = 0.1709), indicating that its apparent diversity was partly attributable to its larger sample size. Because rarefaction to *n* = 2 discards a large proportion of the data, the corrected estimates for the smallest populations (SY and CB) should be interpreted with caution. Overall, these results indicate that *M. azedarach* germplasm from Shaanxi (AK and MX) maintains high genetic diversity, while southern and coastal accessions (JH, DF, and NT) show lower genomic diversity within the collection.

### 3.3. Population Genetic Structure Analysis

The neighbor-joining (NJ) phylogenetic tree divided the 91 *M. azedarach* accessions into two primary lineages (Figure 3). Lineage I split into two sister clades: one exclusively containing the tropical Hainan (HN) subpopulation, and the other clustering the Shaanxi populations from Ankang (AK) and Mianxian (MX). Lineage II included genotypes from Jiangsu (JS), Hubei (HB), and the remaining Shaanxi populations from Zhouzhi (ZZ) and Meixian (ME), showing intertwined clustering patterns among these regions.

Principal component analysis (PCA) showed that the first three principal components explained 30.83% of the total genetic variance (Figure 4). PC1 (19.54%) separated the Hainan island population from all mainland populations along a latitudinal gradient. PC2 (7.29%) separated the coastal Jiangsu population from the inland Shaanxi population. PC3 (4.00%) provided further resolution, placing the Hubei accessions between the Jiangsu and Shaanxi groups.

ADMIXTURE cross-validation (CV) error analysis from *K* = 1 to *K* = 9 showed that the CV error systematically declined and plateaued, reaching a minimum plateau at *K* = 3–4 (CV error 0.44796 and 0.44737, respectively); *K* = 3 was selected as the optimal clustering number on the basis of parsimony (Figure 5). Ancestry clustering profiles from *K* = 2 to *K* = 5 revealed distinct genetic backgrounds among regions (Figure 6). Across *K* models, the Hainan cluster maintained a highly independent and uniform genetic background with no sign of recent introgression. At *K* = 2, the Shaanxi subpopulations from AK and MX formed a distinct genetic component. As *K* increased to *K* = 5, the Jiangsu and Hubei accessions sequentially resolved into separate genetic lineages, while the remaining mainland populations showed mixed ancestral patterns.

To quantify the genetic divergence among geographic regions, pairwise *Fst* values were calculated among the four provinces (Hubei, Hainan, Jiangsu, and Shaanxi; Table 3). The *Fst* between the Hainan island population (Hainan) and the mainland regions was consistently high, ranging from 0.1996 (Hainan vs. Jiangsu) to 0.3670 (Hainan vs. Hubei), whereas the values among the mainland regions were generally lower, ranging from 0.0498 (Hubei vs. Shaanxi) to 0.1998 (HB vs. Jiangsu). The substantially higher *Fst* between the island and the mainland, relative to the within-mainland comparisons, supports the interpretation that the Hainan island has acted as a strong geographic barrier to gene flow.

### 3.4. Isolation-by-Distance (IBD) Analysis

To study spatial genetic patterns, an isolation-by-distance (IBD) analysis was performed using a Mantel test with 999 permutations (Figure 7). The results showed a significant positive correlation between geographic distance (km) and genetic distance among the 91 *M. azedarach* accessions (*r* = 0.307, *p* = 0.001). This significant IBD pattern indicates that greater geographic distance leads to larger genomic divergence. These findings show that despite being preserved under uniform ex situ conditions, these accessions still maintain the spatial genetic differentiation derived from their original wild habitats.

### 3.5. Environmental Association and Redundancy Analysis (RDA)

To analyze the environmental drivers of genetic variation, a redundancy analysis (RDA) was performed at the individual level using two spatial coordinates and seven bioclimatic variables (Figure 8A). The spatial–climatic matrix explained 17.23% of the total genetic variance (F = 2.2804, *p* < 0.001). The first canonical axis (RDA1) explained 8.47% of the variance and was driven primarily by the temperature-related variables bio8 (loading = −0.65) and bio1 (loading = −0.47), separating the tropical Hainan populations from the temperate Shaanxi populations. The second axis (RDA2) accounted for 4.37% of the variance and was governed by the precipitation-related variables bio13 (loading = −0.68) and bio18 (loading = −0.54), separating the humid populations of Hubei and Jiangsu from the semi-arid Shaanxi populations. Based on vector lengths and loadings, the temperature-related variables (bio1 and bio8) and precipitation-related variables (bio13 and bio18) were most strongly associated with the explained genetic variation (Table A2a–c).

Because the individual-level RDA may be influenced by the strong population structure, two complementary analyses were performed (Table A3). In a population-level RDA based on provenance allele frequencies (Figure 8B), the 15 provenances clustered largely by province, and the genetic–environment covariation explained 53.5% of the among-provenance genetic variation, with genetic differentiation strongly coupled to latitude, longitude, and annual mean temperature (bio1). However, in a partial RDA in which population structure (the first five SNP principal components, PC1–PC5) was conditioned out (Figure 8C), the independent contribution of the environmental variables to the residual genetic variation dropped to approximately 4%, and individuals from different provinces overlapped without clear clustering. This contrast indicates that the observed genetic–environment associations are largely driven by geographic isolation and shared ancestry (population structure) rather than by within-population adaptive responses of individuals to local environments.

### 3.6. Genome-Wide Association Analysis of Climate-Related Traits

Both traits showed broad continuous variation with a unimodal distribution across the population (Figure A1), consistent with the polygenic quantitative-trait characteristics expected for such climate variables, and were therefore suitable for genome-wide association analysis. Under the FarmCPU model, 7 significant SNPs were detected for annual mean temperature and 131 for annual precipitation (Table A4). To reduce model-specific false positives, high-confidence loci were defined as the SNPs shared between FarmCPU and another model, which yielded two SNPs for annual mean temperature and one SNP for annual precipitation (Figure 9). Within the 50 kb flanking regions of these shared SNPs, 29 candidate genes were annotated for the two temperature-associated SNPs and 15 candidate genes for the precipitation-associated SNP (Table A5 and Table A6). Genes containing the significant SNP within their body (distance = 0) were prioritized as candidate genes. Accordingly, for annual mean temperature, SNP CM051395.1:18852443 was located within gene OWV82_005272 (18,847,988–18,853,184 bp, plus strand), which encodes a leucine-rich receptor-like protein kinase family protein (NCBI protein accession KAJ4726587.1). For annual precipitation, SNP CM051404.1:2457117 was located within gene OWV82_019844 (2,457,002–2,460,500 bp, plus strand), which encodes a cysteine- and histidine-rich domain-containing protein RAR1 (NCBI protein accessions KAJ4706157.1, KAJ4706158.1, and KAJ4706159.1).

## 4. Discussion

In this study, we estimated the genome-wide population structure, latitudinal diversity gradient, and spatial–climatic associations of *M. azedarach* using 91 accessions from 15 provenances in China. Because these accessions represent open-pollinated maternal half-sib families, our sampling design strictly included only one representative individual per family line. Across the sequence panel, most of the 6,695,869 high-quality SNPs identified were located within non-coding regions (intergenic and intronic zones). This pattern is consistent with genomic landscapes observed in other perennial woody plants and diverse terrestrial taxa, such as *Capsicum annuum* [24], *Camellia* spp. [25], and *Populus tremula* [26], where non-coding regions generally harbor the vast majority of genomic variations. These non-coding regions are widely known to be involved in regulating gene expression and driving phenotypic variation [27,28]. Given the high proportion of intergenic and intronic variants captured here, exploring their potential *cis*-regulatory mechanisms is important for understanding the genetic architecture and adaptive potential of this tree species.

The expected heterozygosity of *M. azedarach* populations was relatively low, but the genome-wide SNP data provided a much higher resolution of genetic variation than the low-density markers used in earlier studies [10]. This relatively low diversity may be linked to limited seed dispersal. As a fleshy-fruited species, *M. azedarach* relies primarily on frugivorous animals, particularly birds, for seed dispersal. However, the drupes contain high concentrations of toxic triterpenoids [29,30], which may deter ingestion by many frugivores [31,32]. Even when fruits are consumed, dispersal can be inefficient: for example, the red-whiskered bulbul (*Pycnonotus jocosus*) in Chinese urban green spaces consumed only the pulp without contributing to seed dispersal [33].

In addition, genetic diversity increased from south to north, from tropical Hainan (~0.11) to subtropical Jiangsu (~0.20) and temperate Shaanxi (~0.28). This differs from the conventional latitudinal trend in temperate woody plants, in which diversity generally decreases with increasing latitude after post-glacial expansion from southern refugia [34,35], and is consistent with [6]. Similar northern-margin patterns have been reported in other temperate trees, such as *Betula platyphylla* and *Picea abies*, where admixture among refugial lineages contributed to high northern diversity [36,37]. The high diversity at the northern margin in *M. azedarach* may similarly reflect the accumulation and mixing of lineages during post-glacial expansion, a contact zone in the Qinling region [34], and selection under the seasonally freezing conditions of the northern margin [38]; however, this inference remains speculative, because the present ex situ dataset cannot disentangle demography, past range shifts, and selection, and no paleo-distribution modelling or wild-population sampling was performed. It should therefore be treated as a hypothesis for future testing.

Phylogenetic reconstruction, PCA, ADMIXTURE clustering (K = 3), and pairwise *Fst* estimates yielded highly congruent results, consistently partitioning the *M. azedarach* collection into distinct geographic lineages, represented by the deep divergence of the Hainan, Shaanxi, and Jiangsu clusters. Notably, all three clustering methods highlighted the tropical Hainan population as a highly uniform genetic cluster with virtually no recent introgression, forming a well-supported monophyletic clade distinct from the mainland gene pools. Pairwise *Fst* confirmed this pattern at the genomic level: the island-mainland *Fst* values (0.1996–0.3670) were markedly higher than those among the mainland provinces (0.0498–0.1998), with the highest value (0.3670) observed between Hainan and Hubei. Hainan is an island separated from the mainland by the Qiongzhou Strait, and this marine barrier appears to have restricted gene flow via pollen or seed dispersal between the island and the mainland [39]. Gene flow acts as a homogenizing force that transfers alleles between populations and counteracts genetic differentiation [40]; when it is reduced or interrupted by a physical barrier, isolated populations can evolve independently through mutation, genetic drift, and local selection, a process termed allopatric divergence [41]. The prolonged isolation of the Hainan population, reflected in its high differentiation from all mainland provinces, has therefore facilitated allopatric divergence, allowing it to accumulate independent variations and evolve into a distinct evolutionary unit. This pattern aligns with findings on other island-mainland plant systems, such as *Epidendrum fulgens* [42], where geographic barriers maintain strong genomic differentiation between mainland and island cohorts.

Concurrently, our results show that the contemporary genetic landscape of *M. azedarach* is governed by a delicate interplay between geographical isolation and environmental filtering. The significant correlation between geographic and genetic distances (*r* = 0.307, *p* = 0.001) supports the classic isolation-by-distance (IBD) model. However, spatial distance alone cannot explain the complete variation. The individual-level RDA showed that bioclimatic variables explained 17.23% of the total genetic variance; however, the complementary partial RDA revealed that, after conditioning on population structure, the independent contribution of the environmental variables fell to approximately 4%. This indicates that most of the genetic–environment covariation is attributable to geographic isolation and shared ancestry, with only a limited independent environmental signal. Consistent with findings in *M. azedarach* [43,44] and sympatric temperate trees [45], the temperature-related variables (bio1 and bio8) and precipitation-related variables (bio13 and bio18) were among those most strongly associated with the explained genetic variation. Collectively, while geographical topology establishes the baseline isolation and largely drives the genetic–environment association, the independent contribution of climatic variables to the adaptive genomic architecture of this species appears limited, and the majority of the genetic variation remains unexplained, likely attributable to historical demography, genetic drift, and unmeasured environmental factors.

The candidate loci identified here provide a complementary line of evidence to the RDA results. Whereas the partial RDA indicated that most of the genetic–environment covariation was attributable to population structure, the mixed-model GWAS accounted for population structure and still detected SNPs significantly associated with annual mean temperature and annual precipitation. These two analyses operate at different levels. The partial RDA quantifies the genome-wide, aggregate variance explained by environment after accounting for population structure, whereas GWAS tests each SNP individually and can detect loci with larger locus-specific effects that are not captured by the aggregate signal. Because the climate variables were derived from provenance locations and are therefore spatially structured, GWAS signals may still partly reflect residual structure and collinearity with demographic history, or alternatively weak polygenic effects. The candidate loci should therefore be regarded as statistical associations rather than confirmed adaptive loci, and functional validation is required before any adaptive role can be claimed. The functional categories of the candidate genes are consistent with an environmental-response interpretation, as leucine-rich receptor-like protein kinases are central components of plant stress signaling [46], and RAR1 participates in defense- and stress-related signaling cascades [47]. These candidates therefore provide promising loci for future investigation of climatic adaptation in *M. azedarach*, rather than demonstrating adaptation directly. Given that they were identified in a single germplasm collection and without functional validation, their actual roles require confirmation through gene-expression analyses and association studies in independent populations.

The ex situ, common-garden design of the germplasm bank provides an important interpretive framework for the climate–genome associations reported here. Because all accessions were propagated from seed and grown under uniform conditions at a single site, environmental plasticity, that is, within-generation phenotypic adjustment to local conditions, could not confound the associations between genetic variants and the climate variables of the source provenances. Consequently, the genetic-environment correlations we detected are best interpreted as consistent with historical adaptation to the environments of the original collection sites rather than contemporary plastic responses [17,48]. At the same time, this design cannot capture the effects of the current in situ environment, and our results therefore describe the genetic signature of past environmental association preserved in the conserved germplasm rather than the ongoing adaptive response of wild populations. These considerations support the interpretation of the IBD and RDA results as evidence of historical spatial and climatic differentiation, while underscoring that direct inferences about contemporary adaptation would require additional data from wild populations or common-garden experiments.

Several limitations should be acknowledged. Because the accessions derive from open-pollinated maternal half-sib families, residual paternal relatedness cannot be fully excluded. Provenance sample sizes were uneven (2–10 individuals) and may still affect ADMIXTURE, PCA, *Fst*, and RDA despite the rarefaction applied to diversity statistics. In addition, the bioclimatic variables represent provenance-level macroclimate derived from origin coordinates, so environmental variation within provenances is not captured and the genotype–environment associations may be attenuated rather than inflated. Finally, only a single ADMIXTURE run was performed for each K value, so the results may be sensitive to the starting conditions.

## 5. Conclusions

This study decoded the population genomic landscape of 91 *M. azedarach* accessions, revealing distinct geographic structure, a northward-increasing diversity gradient, and a highly independent Hainan population isolated across the Qiongzhou Strait. These findings, together with the climate-associated candidate loci identified through genome-wide association analysis, provide a genetic basis for conserving the germplasm in the National Germplasm Bank, particularly the high-diversity Shaanxi provenances and the distinct Hainan population. Because all accessions were conserved ex situ, our results primarily reflect the conserved germplasm rather than contemporary wild populations. In addition, the interpretation of northern high-diversity regions as refugial or contact zones remains speculative without paleo-distribution modelling or wild-population sampling. Future work should combine population genomic scans with functional validation in independent populations to test the candidate loci potentially underlying climatic adaptation.

## Figures and Tables

**Figure 1:**
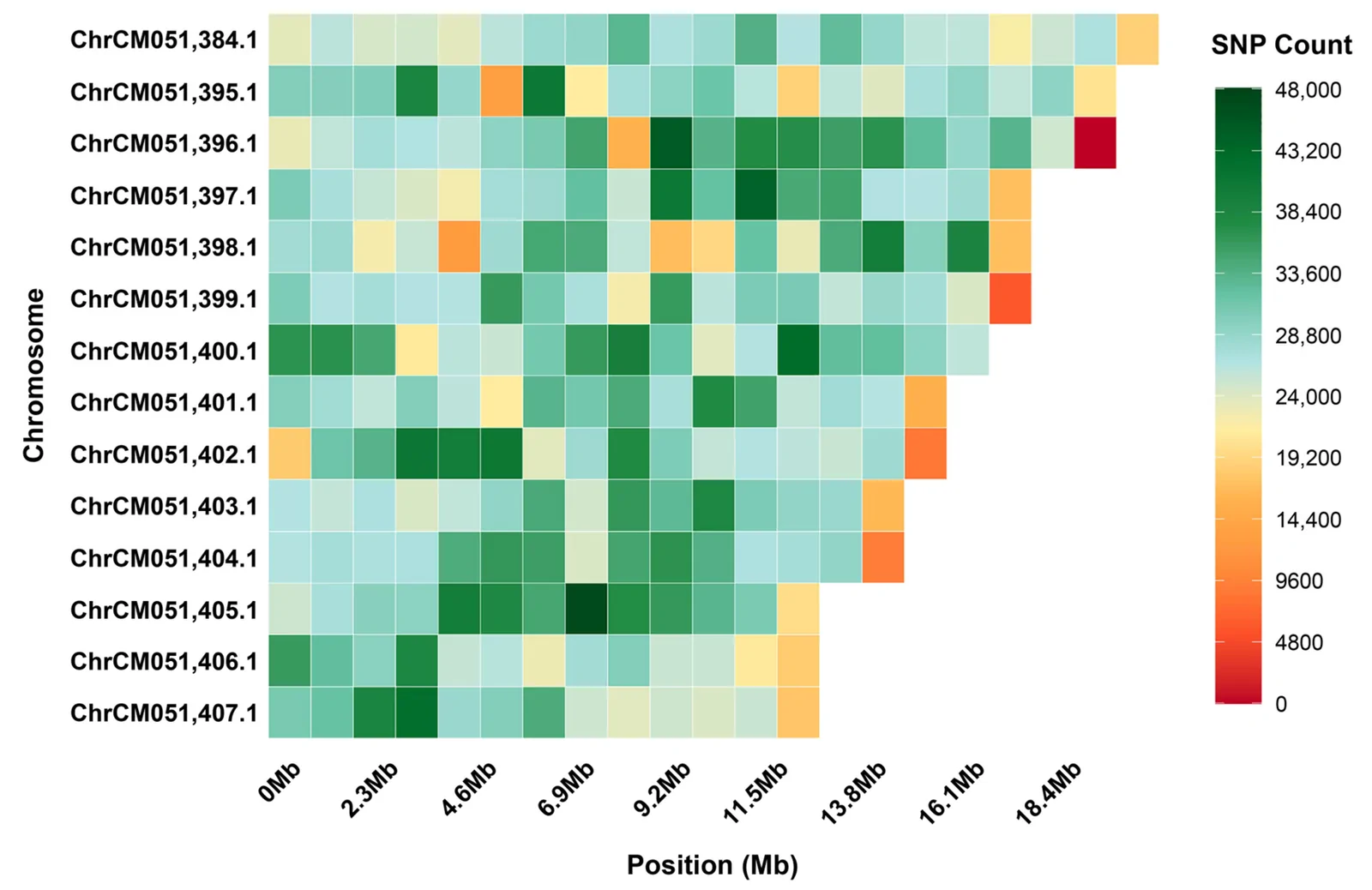
Chromosomal distribution of 6,695,869 SNPs across the 14 pseudochromosomes of *M. azedarach.* Color gradient (red to dark green) indicates SNP density per 1-Mb window.

**Figure 2:**
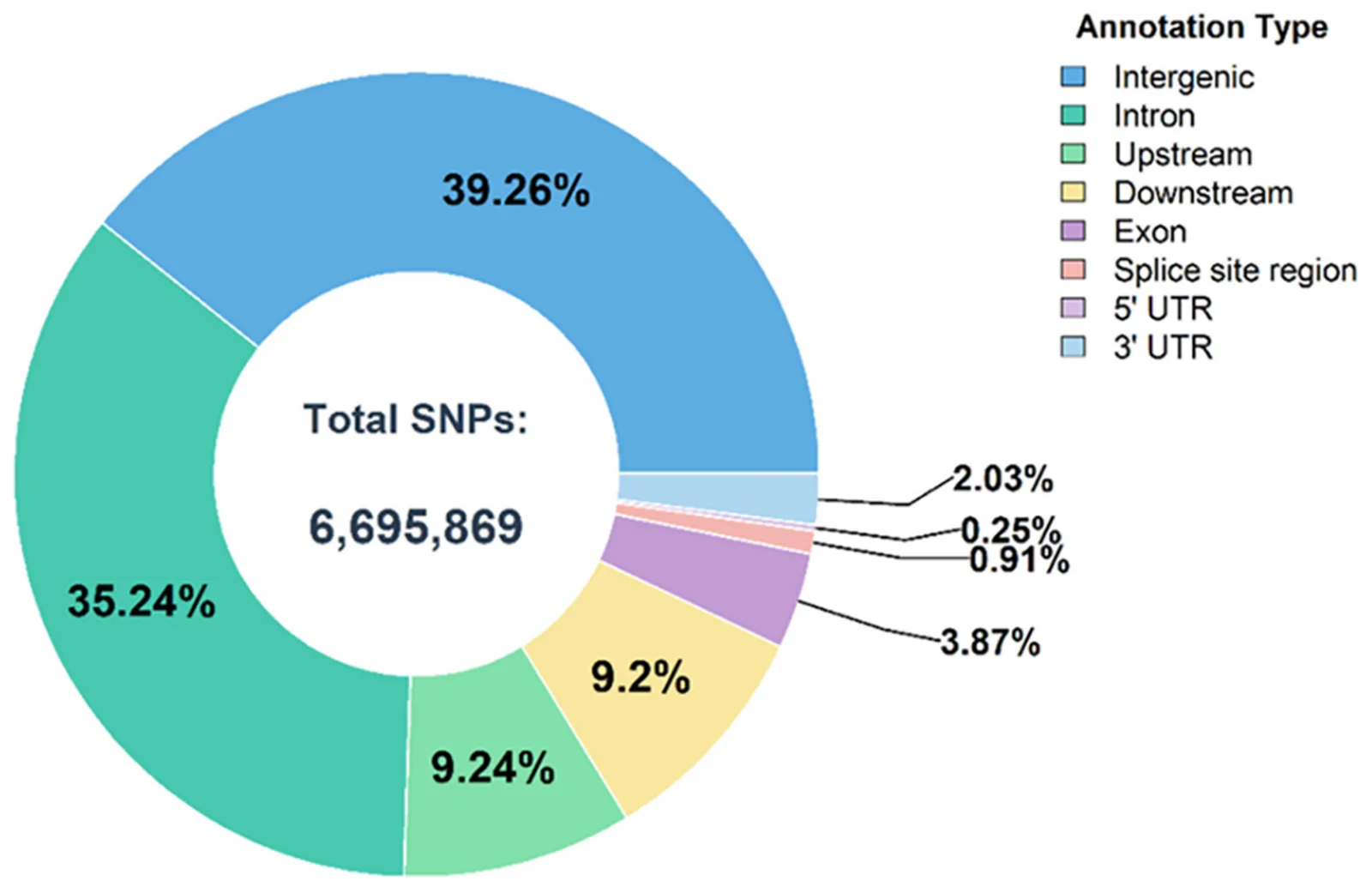
Functional annotation of the identified SNPs across genomic regions.

**Figure 3:**
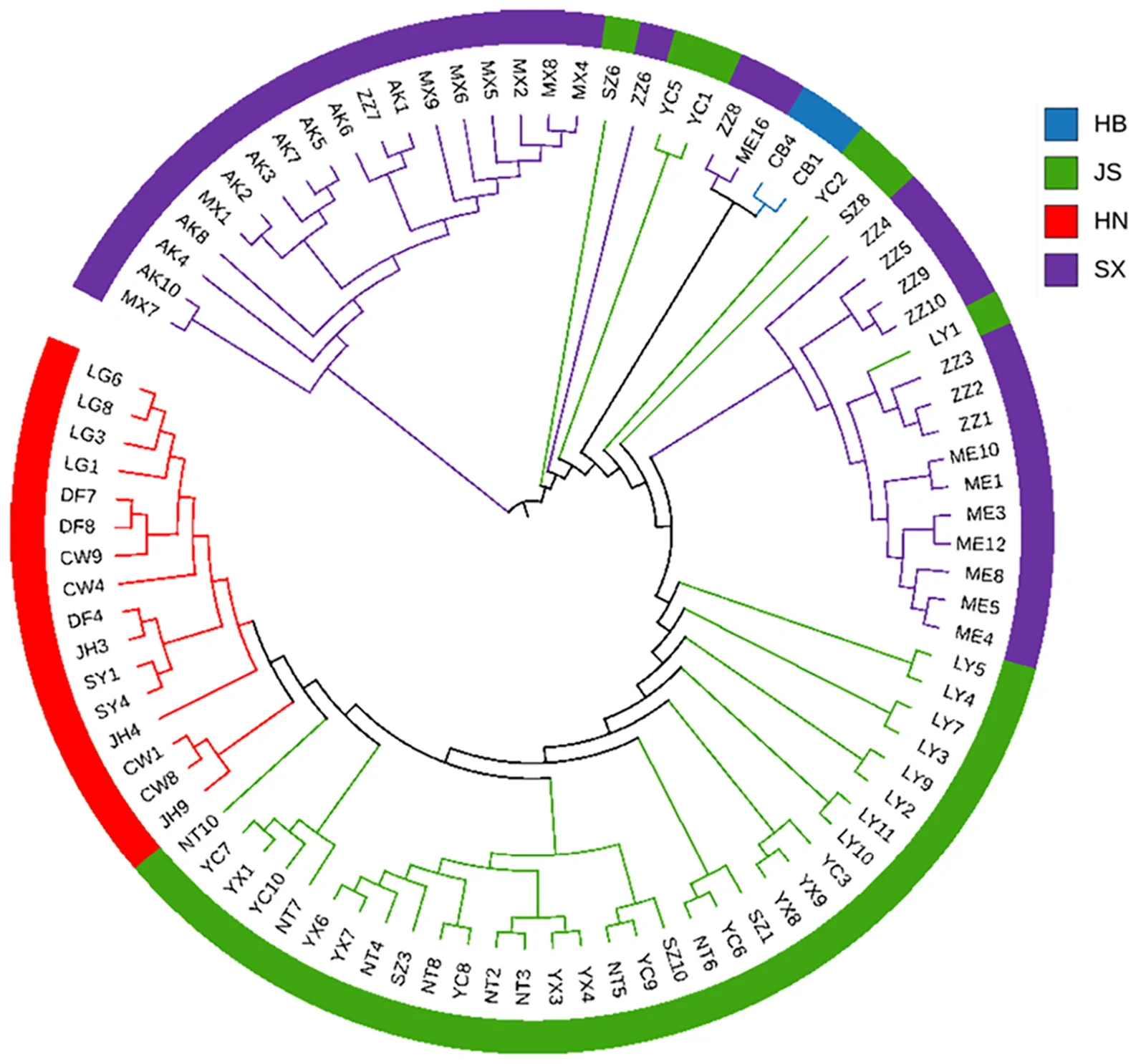
Neighbor-joining phylogenetic tree of the 91 *M. azedarach* germplasm accessions based on core genomic SNPs. Colored rings indicate four geographic origins: HB (Hubei), HN (Hainan), JS (Jiangsu), and SX (Shaanxi). The same abbreviations apply to subsequent figures.

**Figure 4:**
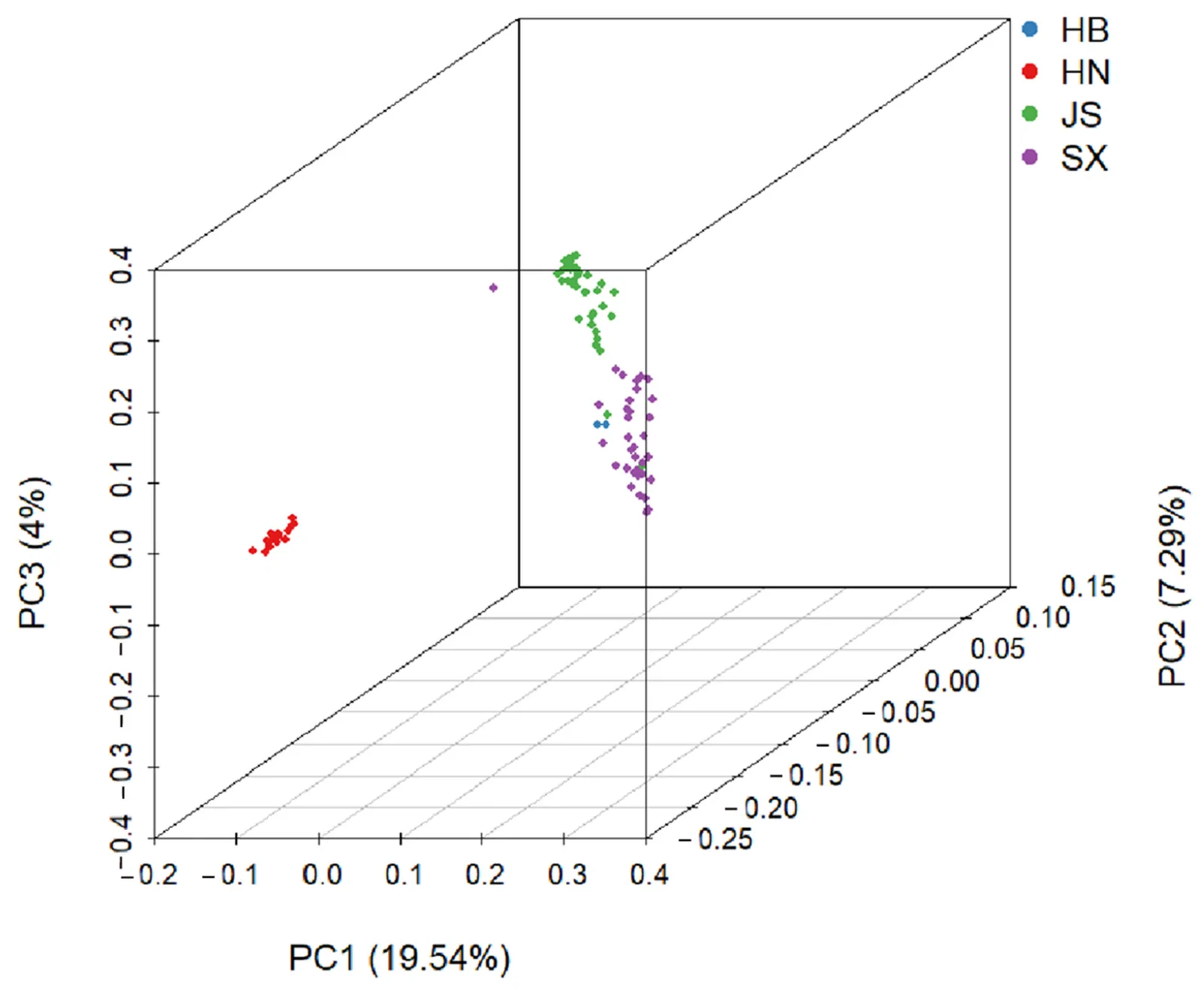
Principal component analysis of 91 *M. azedarach* individuals across the first three principal components (PC1–PC3). Colors denote geographic origins.

**Figure 5:**
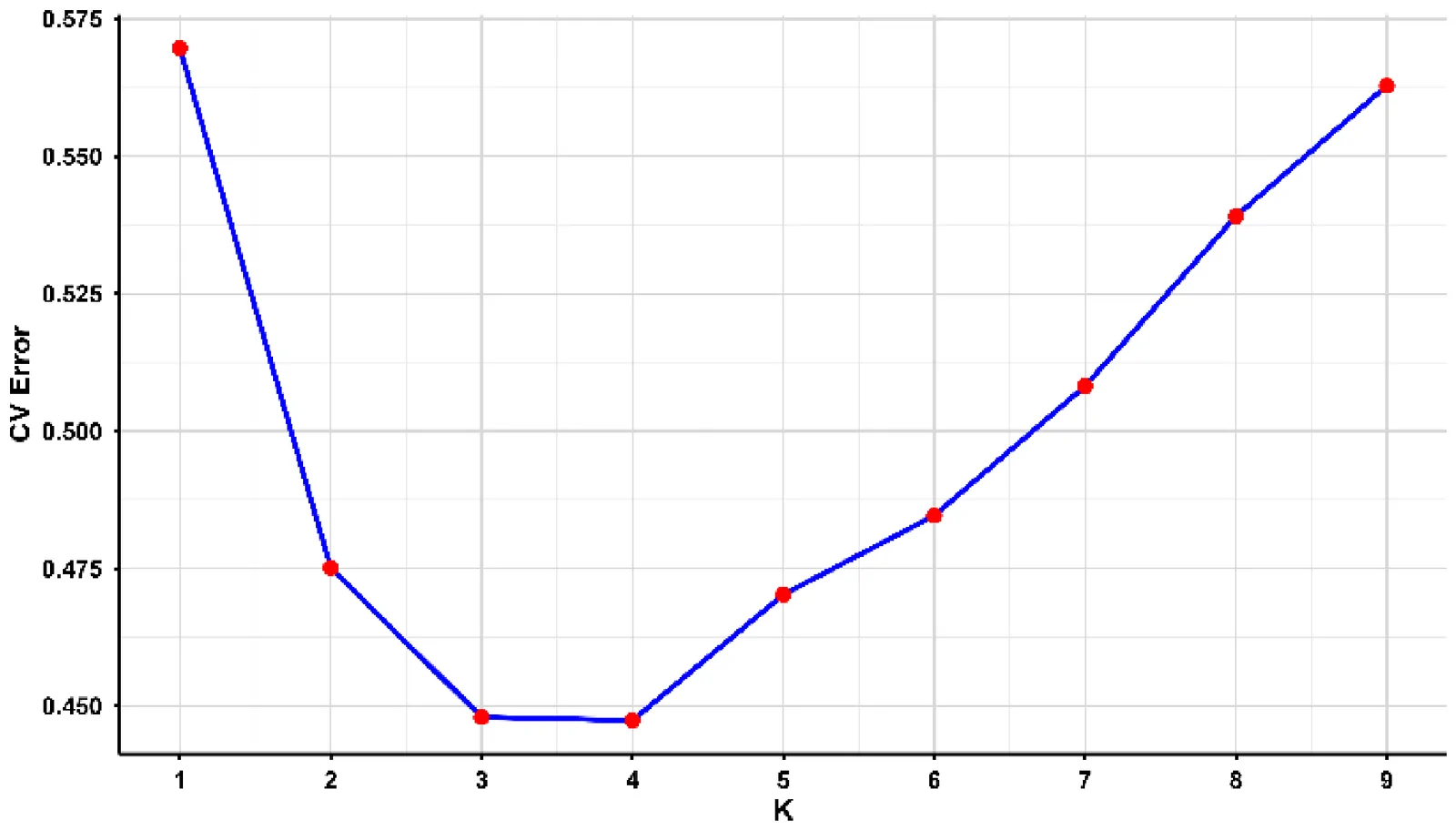
Cross-validation (CV) error scores for ADMIXTURE clustering from *K* = 1 to *K* = 9. Red dots represent the cross‑validation error for each tested *K*; the blue line indicates the fitted curve. The optimal *K * was determined at the minimum cross‑validation error.

**Figure 6:**
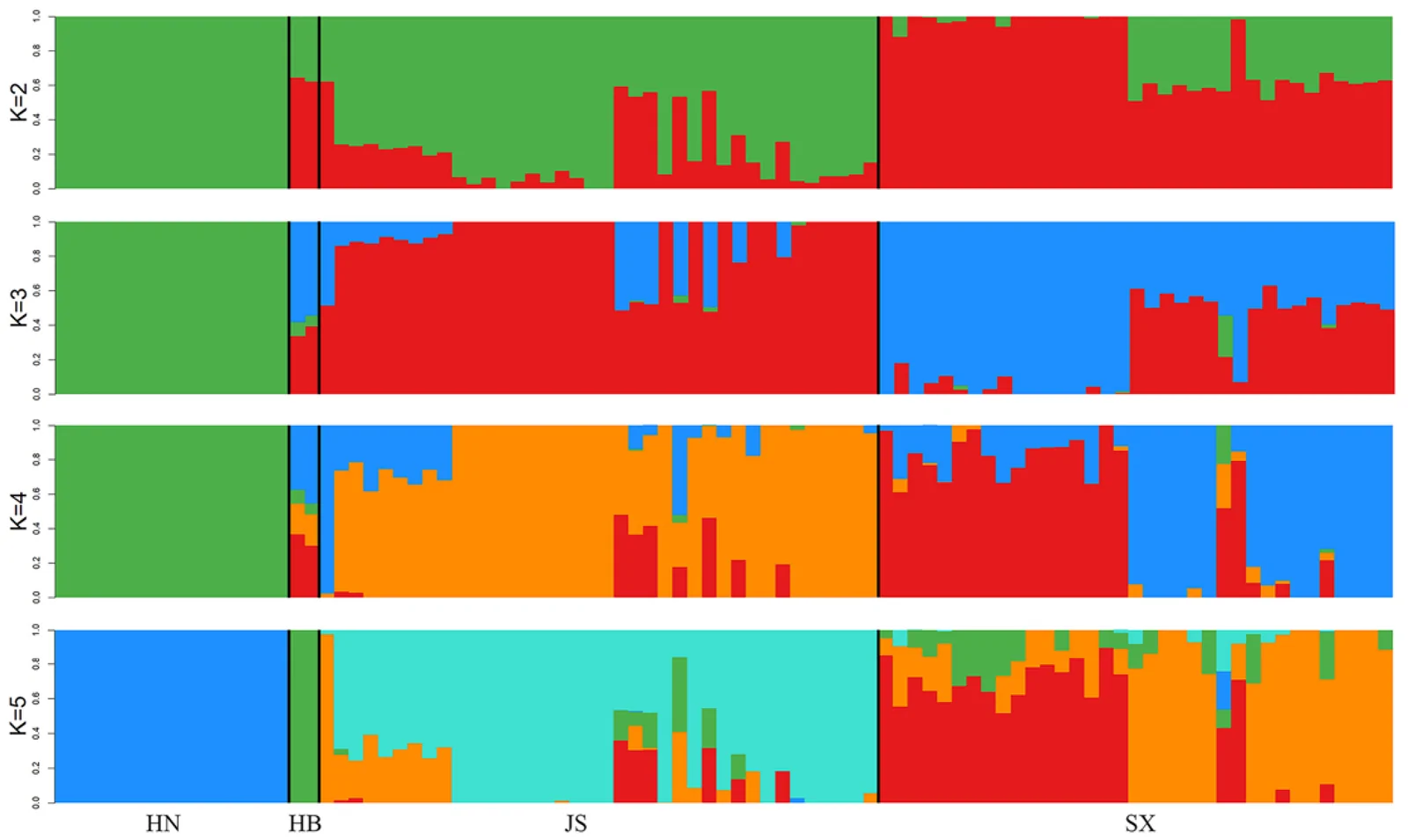
Population genetic structure inferred by ADMIXTURE at *K* = 2 to *K* = 5. Black vertical lines separate the four province-geographic groups (HB, Hubei; HN, Hainan; JS, Jiangsu; SX, Shaanxi). Each vertical bar represents one individual. Different colors indicate distinct genetic clusters. The individuals are arranged according to their sampling populations.

**Figure 7:**
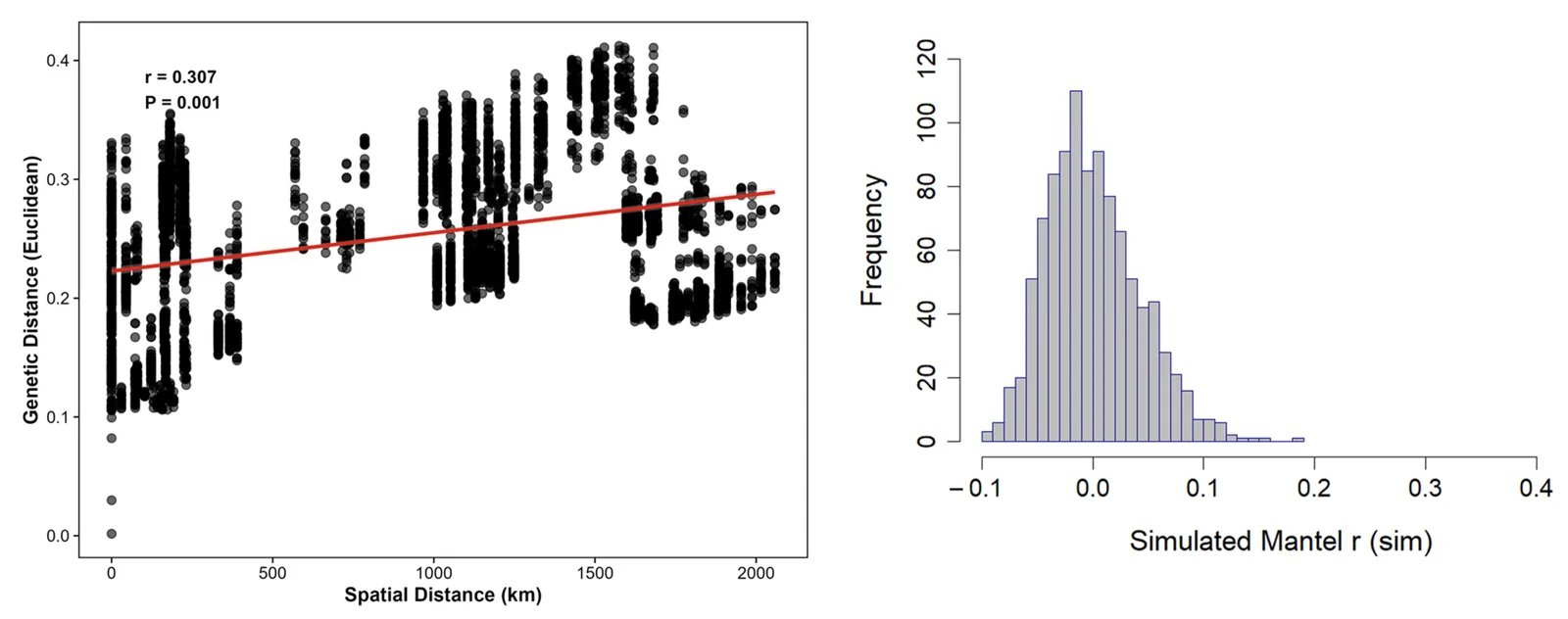
Isolation-by-distance (IBD) analysis based on the Mantel test with 999 permutations. (**Left**): linear regression of genetic distance against geographic distance; the red line denotes the regression line. (**Right**): null distribution of correlation coefficients from 999 permutations; red line indicates observed *r* = 0.307 (*p* = 0.001).

**Figure 8:**
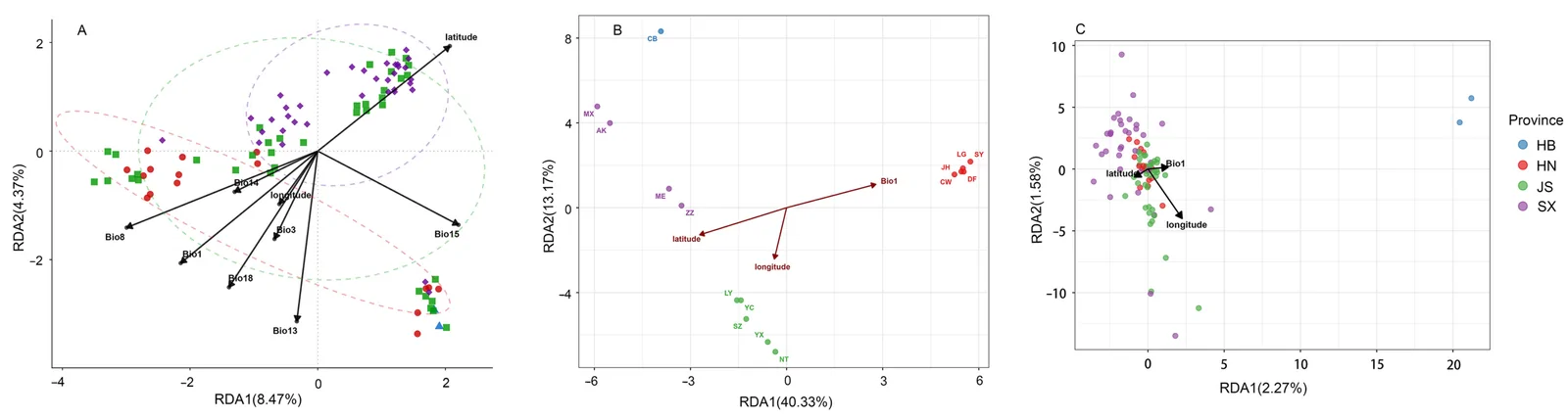
Redundancy analysis (RDA) of genomic variation with environmental variables in the 91 *M. azedarach* accessions. (**A**) Individual-level RDA based on all 91 accessions, two spatial coordinates, and seven bioclimatic variables; (**B**) population-level RDA based on provenance allele frequencies and three environmental variables (longitude, latitude, and annual mean temperature, bio1); (**C**) partial RDA in which population structure (first five SNP principal components) was conditioned out. Dots are colored by province (HN, Hainan; JS, Jiangsu; SX, Shaanxi; HB, Hubei), and blue arrows indicate the environmental vectors.

**Figure 9:**
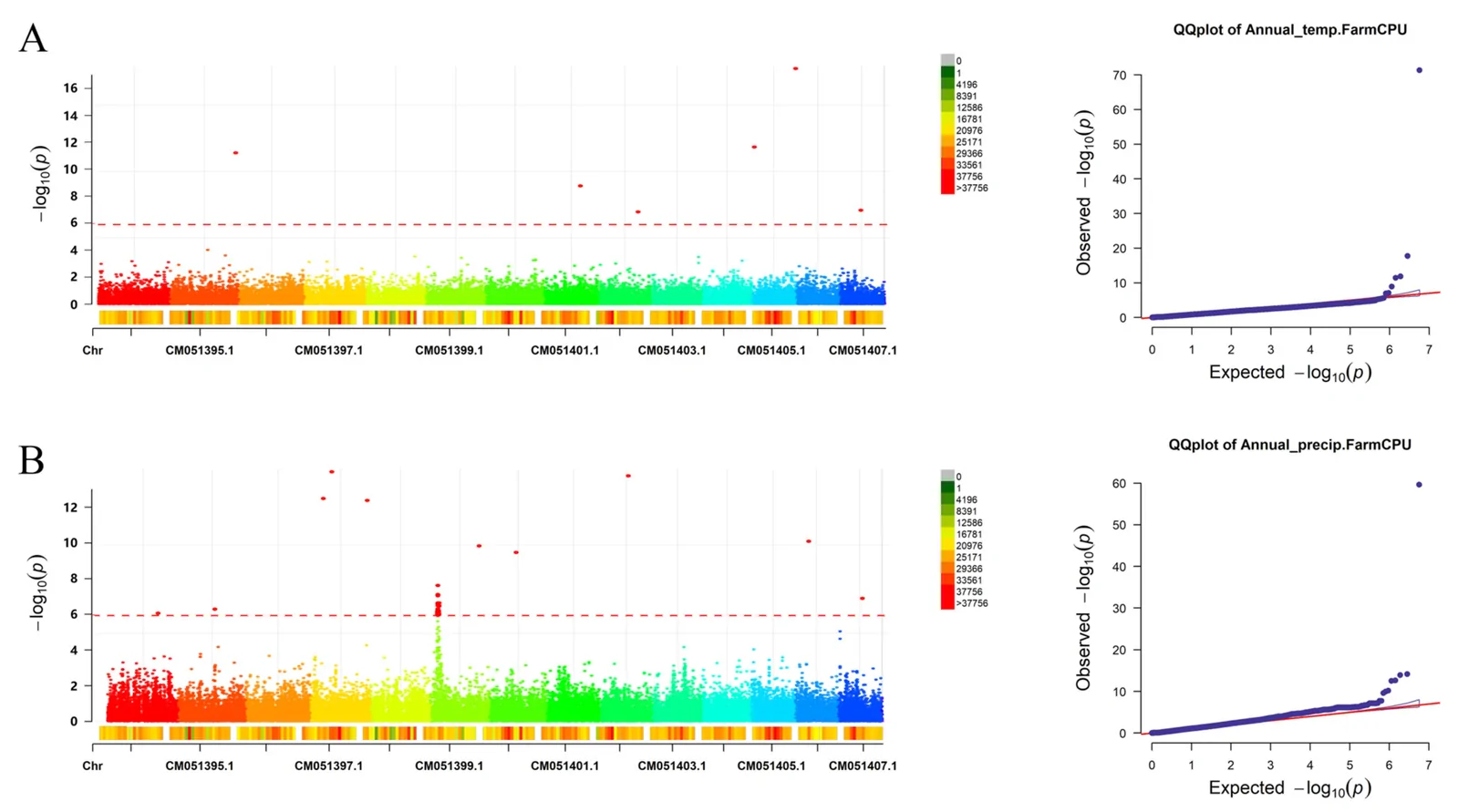
Genome-wide association analysis of the two climate-related traits based on the FarmCPU model. (**A**) Annual mean temperature; (**B**) annual precipitation. Each dot represents an SNP, and the horizontal line indicates the significance threshold (−log_10_(P) ≥ 6).

**Table 1:** Geographic origins of the 91 investigated *M. azedarach* accessions in China.

Province	Provenance	Longitude	Latitude	Sample Size *	Elevation (m)
Hainan	Lingao (LG)	109°45′08” E	19°52′27” N	4	56
Hainan	Dongfang (DF)	108°39′39” E	19°06′46” N	3	100
Hainan	Chengmai (CW)	110°00′10” E	19°44′26” N	4	60
Hainan	Qionghai (JH)	110°28′51” E	19°15′33” N	3	120
Hainan	Sanya (SY)	109°30′29” E	18°15′39” N	2	60
Hubei	Chibi (CB)	113°54′40” E	29°43′35” N	2	90
Jiangsu	Lianyungang (LY)	119°13′07” E	34°36′24” N	9	50
Jiangsu	Nantong (NT)	120°53′12” E	31°58′43” N	8	105
Jiangsu	Suzhou (SZ)	120°35′36” E	31°18′27” N	5	28
Jiangsu	Yancheng (YC)	120°09′12” E	33°21′16” N	9	60
Jiangsu	Yixing (YX)	119°49′30” E	31°20′55” N	7	75
Shaanxi	Ankang (AK)	109°01′58” E	32°41′24” N	9	80
Shaanxi	Mianxian (MX)	106°40′48” E	33°09′09” N	8	60
Shaanxi	Zhouzhi (ZZ)	108°13′39” E	34°09′42” N	10	45
Shaanxi	Meixian (ME)	107°45′34” E	34°16′27” N	8	106

* Note: Each sampled individual represents a single, independent family accession preserved in the National Germplasm Resource Bank of *M. azedarach*. To eliminate pedigree-relatedness bias, only one representative plant per family was sequenced to avoid genetic bias.

**Table 2:** Genetic diversity parameters of the studied *M. azedarach* populations.

Provenance	*N_e_*	*H_o_*	*H_e_*	PIC	*π*	*N_e_ ^†^	*H_e_* ^†^
LG	1.2004	0.1413	0.1183	0.0949	0.1370	1.0587	0.0587
DF	1.1779	0.1335	0.1038	0.0825	0.1281	1.0568	0.0579
CW	1.2018	0.1417	0.1194	0.0959	0.1383	1.0563	0.0583
JH	1.1870	0.1299	0.1093	0.0871	0.1340	1.0632	0.0646
SY	1.1666	0.1375	0.0974	0.0765	0.1351	1.0669	0.0673
CB	1.3483	0.3246	0.1999	0.1559	0.2867	1.1774	0.1818
LY	1.3420	0.2318	0.2106	0.1722	0.2251	1.105	0.1073
NT	1.2460	0.1719	0.1539	0.1268	0.1657	1.0528	0.0541
SZ	1.3493	0.2593	0.2184	0.1795	0.2455	1.1442	0.1455
YC	1.3710	0.2597	0.2346	0.1939	0.2504	1.1587	0.1619
YX	1.2776	0.2118	0.1717	0.1408	0.1869	1.1793	0.1850
AK	1.5790	0.3711	0.3326	0.2640	0.3557	1.2357	0.2400
MX	1.5825	0.3909	0.3316	0.2622	0.3584	1.1653	0.1709
ZZ	1.4191	0.2688	0.2543	0.2067	0.2718	1.1923	0.1982
ME	1.3617	0.2438	0.2172	0.1758	0.2356	1.0914	0.0948

* Note: ^†^ Indicates rarefaction-corrected data (minimum sample size *n* = 2). *N_e_*^†^ and *H_e_*^†^ are derivative parameters recalculated based on the rarefied dataset.

**Table 3:** Pairwise *Fst* matrix among the four provinces.

	Hubei	Hainan	Jiangsu	Shaanxi
Hubei	—	0.3670	0.1998	0.0498
Hainan	0.3670	—	0.1996	0.2615
Jiangsu	0.1998	0.1996	—	0.1429
Shaanxi	0.0498	0.2615	0.1429	—

**Figure A1:**
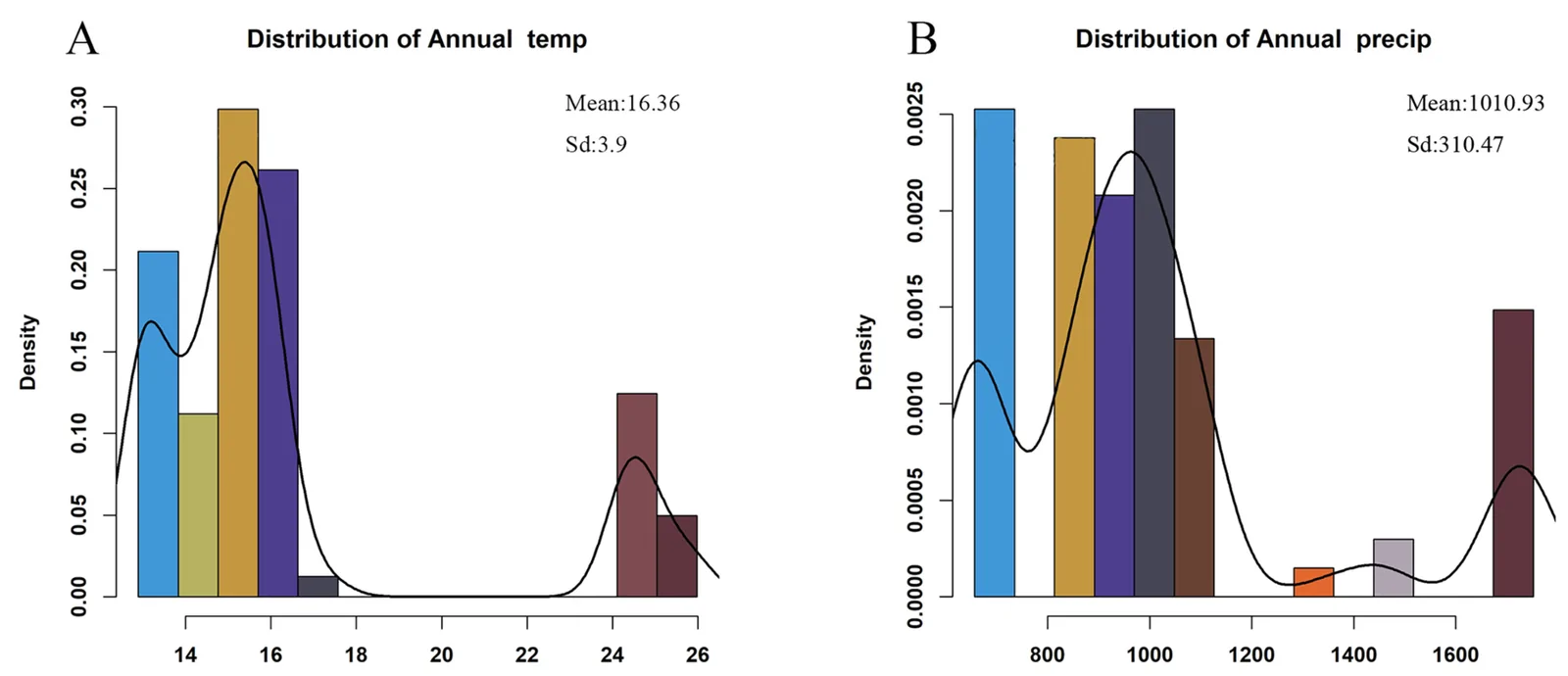
Frequency-distribution histograms of the two climate-related traits across the 91 *M. azedarach* accessions. (**A**) Annual mean temperature (bio1); (**B**) annual precipitation (bio13).

## Data Availability

The raw sequencing data and SNP dataset generated in this study will be deposited in the NCBI Sequence Read Archive (SRA) and dbSNP databases upon acceptance of the manuscript. Accession numbers will be provided at that time. The raw paired-end whole-genome resequencing data generated in this study have been deposited in the Sequence Read Archive (SRA) at the National Center for Biotechnology Information (NCBI) under BioProject accession number PRJNAXXXXXX. All datasets are currently restricted during the peer-review process and will be made fully and permanently accessible to the public immediately upon the formal acceptance of this manuscript.

## Appendix A

**Table A1:** Sequencing quality control statistics for each *M. azedarach* provenance.

Provenance	Sample Size	Mean Sequencing Depth	Mapping Rate (%)	Genome Coverage (%)	Missing Rate (%)	Ti/Tv Ratio
LG	4	11.69	94.90	91.37	5.93	2.09
DF	3	11.47	94.25	89.70	7.53	2.10
CW	4	12.58	94.08	90.25	7.01	2.10
JH	3	12.37	94.94	91.99	5.24	2.08
SY	2	12.03	95.43	90.52	6.65	2.13
CB	2	11.31	93.96	88.27	9.07	2.04
LY	9	11.93	95.01	88.40	9.03	2.06
NT	8	11.41	92.44	89.07	8.37	2.06
SZ	5	11.69	94.77	90.86	6.51	2.04
YC	9	11.72	95.15	88.91	8.45	2.05
YX	7	11.37	94.30	87.96	9.55	2.07
AK	9	11.62	93.98	86.41	11.02	2.01
MX	8	11.66	94.17	87.41	9.76	2.05
ZZ	10	11.14	89.23	79.91	17.77	2.03
ME	8	11.82	93.14	86.10	11.36	2.07

**Table A2:** Summary of the individual-level redundancy analysis (RDA). (a) Eigenvalues and proportion of variance explained by the first five axes of redundancy analysis (RDA); (b) significance test of the RDA model based on permutation tests; (c) Scores (loadings) of environmental variables on the first two RDA axes.

**(a)**				
**RDA**	**Eigenvalue**	**Proportion Explained (%)**	**Cumulative Proportion (%)**	
RDA1	18.44	8.47	8.47	
RDA2	9.50	4.37	12.84	
RDA3	4.09	1.88	14.72	
RDA4	2.85	1.31	16.03	
RDA5	2.61	1.20	17.23	
Total explained variation	37.49	17.23		
**(b)**				
	**Df**	**Variance**	**F**	***p***
RDA Model	9	43.984	2.2804	0.001
Residual	81	173.591		
**(c)**				
	**RDA1**		**RDA2**	
Longitude	−0.1306		−0.2117	
Latitude	0.4483		0.4195	
bio1	−0.4654		−0.4475	
bio3	−0.1468		−0.3509	
bio8	−0.6490		−0.3058	
bio13	−0.0712		−0.6800	
bio14	−0.2823		−0.1627	
bio15	0.4776		−0.2946	
bio18	−0.3014		−0.5438	

**Table A3:** Comparison of the three redundancy analysis (RDA) models for genomic variation with environmental variables.

RDA Model	Analysis Unit	Conditioning Variables	Environmental Variables	Explained Variance (%)	F	*p*
Individual-level	91 accessions	None	2 spatial + 7 bioclimatic	17.23	2.2804	<0.001
Population-level	15 provenances	None	longitude, latitude, bio1	53.5	6.5255	<0.001
Partial	91 accessions	PC1–PC5	longitude, latitude, bio1	~4	1.495	<0.001

**Table A4:** Number of significant SNPs detected for the two climate-related traits under the three GWAS models.

Trait	Model	No. of Significant SNPs
Annual mean temperature	GLM	917,804
Annual mean temperature	MLM	0
Annual mean temperature	FarmCPU	7
Annual precipitation	GLM	908,549
Annual precipitation	MLM	4784
Annual precipitation	FarmCPU	131

**Table A5:** Candidate genes associated with annual mean temperature based on FarmCPU.

Gene_ID	Start	End	Strand	Distance to SNP	Product	Protein_IDs
OWV82_005265	18804398	18807813	+	44630	Protein kinase superfamily protein	KAJ4726578.1
OWV82_005266	18813588	18818900	+	33543	DnaJ domain-containing protein	KAJ4726579.1; KAJ4726580.1
OWV82_005267	18819508	18824087	+	28356	Endoplasmic reticulum oxidoreductin-1	KAJ4726581.1; KAJ4726582.1
OWV82_005268	18824158	18829386	-	23057	Kinesin-like protein	KAJ4726583.1
OWV82_005269	18829623	18836815	-	15628	Translation initiation factor IF-2	KAJ4726584.1
OWV82_005270	18837264	18839721	-	12722	Release factor glutamine methyltransferase	KAJ4726585.1
OWV82_005271	18845458	18847250	+	5193	Protein kinase superfamily protein	KAJ4726586.1
OWV82_005272	18847988	18853184	+	0	Leucine-rich receptor-like protein kinase family protein	KAJ4726587.1
OWV82_005273	18854246	18855690	+	1803	putative Glutathione s-transferase	KAJ4726588.1
OWV82_005274	18863128	18866133	+	10685	Glutathione S-transferase	KAJ4726589.1
OWV82_005275	18867982	18869004	+	15539	Glutathione S-transferase	KAJ4726590.1
OWV82_005276	18874906	18876698	+	22463	Glutathione S-transferase	KAJ4726591.1
OWV82_005277	18879787	18880847	+	27344	Glutathione S-transferase	KAJ4726592.1
OWV82_005278	18882976	18884145	+	30533	putative Glutathione s-transferase	KAJ4726593.1
OWV82_005279	18884292	18886340	-	31849	CASP-like protein	KAJ4726594.1
OWV82_005280	18887151	18889567	-	34708	Peptide-methionine (R)-S-oxide reductase	KAJ4726595.1; KAJ4726596.1
OWV82_005281	18889768	18894818	+	37325	Ribokinase	KAJ4726597.1
OWV82_005282	18895517	18896252	+	43074	hypothetical protein	KAJ4726598.1
OWV82_005283	18896410	18897370	-	43967	Glutathione S-transferase	KAJ4726599.1
OWV82_021219	601446	603311	-	47929	Peroxidase	KAJ4704286.1
OWV82_021220	606356	608485	-	42755	Peroxidase	KAJ4704287.1
OWV82_021221	625156	626950	-	24290	transcription factor SCREAM2-like	KAJ4704288.1; KAJ4704289.1
OWV82_021222	634222	636140	-	15100	G-type lectin S-receptor-like serine/threonine-protein kinase	KAJ4704290.1
OWV82_021223	642121	643015	+	8225	Alanine--glyoxylate aminotransferase 2-like 2 mitochondrial	KAJ4704291.1
OWV82_021224	652394	654802	+	1154	Alpha-ketoglutarate-dependent dioxygenase AlkB	KAJ4704292.1; KAJ4704293.1; KAJ4704294.1
OWV82_021225	656941	663296	+	5701	Alanine--glyoxylate aminotransferase 2, mitochondrial-like protein	KAJ4704295.1
OWV82_021226	671985	674954	+	20745	Alanine--glyoxylate aminotransferase 2, mitochondrial-like protein	KAJ4704296.1
OWV82_021227	685587	688523	+	34347	Alanine--glyoxylate aminotransferase 2, mitochondrial-like protein	KAJ4704297.1
OWV82_021228	693908	697510	-	42668	Alanine--glyoxylate aminotransferase 2, mitochondrial-like protein	KAJ4704298.1

**Table A6:** Candidate genes associated with annual precipitation based on FarmCPU.

Gene_ID	Start	End	Strand	Distance to SNP	Product	Protein_IDs
OWV82_019836	2407803	2410761	-	46356	Cytochrome P450 family protein	KAJ4706147.1
OWV82_019837	2416031	2418041	-	39076	Pentatricopeptide repeat-containing protein	KAJ4706148.1
OWV82_019838	2418255	2422197	-	34920	Mitochondrial metalloendopeptidase OMA1	KAJ4706149.1
OWV82_019839	2423146	2428141	-	28976	DEAD-box ATP-dependent RNA helicase	KAJ4706150.1
OWV82_019840	2435337	2437461	-	19656	Embryo-specific	KAJ4706151.1
OWV82_019841	2438940	2441641	-	15476	Xyloglucan galactosyltransferase	KAJ4706152.1; KAJ4706153.1
OWV82_019842	2445354	2447451	-	9666	CDGSH iron-sulfur domain-containing protein NEET	KAJ4706154.1; KAJ4706155.1
OWV82_019843	2447870	2456264	+	853	K(+) efflux antiporter	KAJ4706156.1
OWV82_019844	2457002	2460500	+	0	cysteine and histidine-rich domain-containing protein RAR1	KAJ4706157.1; KAJ4706158.1; KAJ4706159.1
OWV82_019845	2464342	2470769	+	7225	Receptor protein kinase	KAJ4706160.1
OWV82_019846	2471764	2477191	-	14647	protein TPX2-like	KAJ4706161.1; KAJ4706162.1
OWV82_019847	2480412	2483033	+	23295	1-aminocyclopropane-1-carboxylate synthase	KAJ4706163.1
OWV82_019848	2482859	2484308	-	25742	putative Hydroxyproline-rich glycoprotein family protein	KAJ4706164.1
OWV82_019849	2489348	2490193	+	32231	Zinc finger A20 and AN1 domain-containing stress-associated protein	KAJ4706165.1
OWV82_019850	2496182	2497912	-	39065	DUF668 domain-containing protein/DUF3475 domain-containing protein	KAJ4706166.1

## References

[1] Cai J.-F., Yang X.-M., Yu W.-W., Wang G.-B., Cao F.-L. (2021). Development of SSR molecular markers based on transcriptome sequencing of *Melia azedarach*. Sci. Silvae Sin..

[2] Zhang X.-Y., Wang X.-L., Yuan H., Su J., Wu Y.-B. (2024). Research progress on chemical constituents and pharmacological activities of *Melia azedarach*. J. Chin. Med. Mater..

[3] Liao Y.-L., Cai S.-Z., Ye C., Liu M.-S., Xu X.-M., Tao R. (2015). Effects of simulated acid rain on ecophysiological characteristics of *Melia azedarach*. Chin. J. Ecol..

[4] Zhao B., Cheng M., Xue Y.-B., Zhang J.-H., Zhang Z.-H., Pan X.-B., Wang Y.-L. (2023). Research progress on chemical constituents, pharmacological activities and clinical application of *Melia cortex*. West China J. Pharm. Sci..

[5] He X., Deng C., Yang J.-Q., Zhang D., Zhang M.-J., Liao B.-Y., Wang F., Chen X.-Y. (2018). Geographic variation of growth traits in early stage for *Melia azedarach* among different provenances. J. Beijing For. Univ..

[6] Cheng S.-M., Gu W.-C., Shi X.-D. (2024). Coupling analysis on genetic diversity of *Melia azedarach*. Mol. Plant Breed..

[7] Huang Y.-Y., Gao E.-J., Yuan J.-Q., Wu Y.-C., Rao X.-P., Wang W. (2026). A new limonoid compound from the bark of *Melia azedarach* and its anti-hepatocarcinoma activity. Acta Pharm. Sin..

[8] Cheng S.-M., Gu W.-C. (2006). Studies on phenotypic allocation of *Melia azedarach*. For. Res..

[9] Chen L.-J., Deng X.-M., Ding M.-M., Liu M.-Q., Li J.-C., Hui W.-K., Liao B.-Y., Chen X.-Y. (2014). Geographic variation in traits of fruit stones and seeds of *Melia azedarach*. J. Beijing For. Univ..

[10] Liao B.-Y., Wang F., Chen L.-J., Liu M.-Q., Ouyang K.-X., Que Q.-M., Hui W.-K., Li P., Chen X.-Y. (2016). Genetic diversity of germplasm resources of *Melia azedarach* revealed by SRAP markers. Sci. Silvae Sin..

[11] Wan X.-Y., Xu M.-Y., Ye X.-M., Zhong X.-R., Ye X.-Y., Wu N.-S., Sun R.-X. (2024). Research on genetic diversities of *Melia azedarach* populations in Jiangxi. Guangxi For. Sci..

[12] Cai J.-F., Yang X.-M., Yu W.-W., Xiang P., Zhang S.-Q., Wang G.-B. (2022). The diversity of *Melia azedarach* L. from China based on transcriptome-developed SSR marker. Forests.

[13] Liao X.-L., Gu L.-T., Li B., Liu W., Xu Z.-B., Wei Z.-H., Zhai J.-T., Xu F., Tang J., Guo Y. (2026). Genetic variation patterns in the whole genome of hybrid germplasm between *Populus* sect. *Aigeiros* and *Populus* sect. *Tacamahaca*. J. Northeast. For. Univ..

[14] Chen X.-Z. (2025). Whole-Genome Resequencing Analysis of Genetic Structure and Population Demographic History of *Prunus cerasoides*. Master’s Thesis.

[15] Wang J., Hu Z.-B., Liao X.-L., Wang Z.-Y., Li W., Zhang P.-P., Cheng H., Wang Q., Bhat J.A., Wang H. (2022). Whole-genome resequencing reveals signature of local adaptation and divergence in wild soybean. Evol. Appl..

[16] Sajjad M., Ahmad A., Riaz M.W., Hussain Q., Yasir M., Lu M.-Z. (2023). Recent genome resequencing paraded COBRA-like gene family roles in abiotic stress and wood formation in poplar. Front. Plant Sci..

[17] Xiao Z.-Q., Yang T., Wang S.-T., Wei X.-Z., Jiang M.-X. (2021). Exploring the origin and genetic representation of ex situ living collections of five endangered tree species established for 20–35 years. Glob. Ecol. Conserv..

[18] Walters C., Pence V.C. (2021). The unique role of seed banking and cryobiotechnologies in plant conservation. Plants People Planet.

[19] Lee H. (2023). Ex situ conservation efforts for plant diversity protection with a focus on seeds. Open Agric. J..

[20] Breman E., Ballesteros D., Castillo-Lorenzo E., Cockel C., Dickie J., Faruk A., O’donnell K., Offord C.A., Pironon S., Sharrock S. (2021). Plant diversity conservation challenges and prospects—The perspective of botanic gardens and the Millennium Seed Bank. Plants.

[21] Salgotra R.K., Chauhan B.S. (2023). Genetic diversity, conservation, and utilization of plant genetic resources. Genes.

[22] Doyle J.J., Doyle J.L. (1987). A rapid DNA isolation procedure for small quantities of fresh leaf tissue. Phytochem. Bull..

[23] Forester B.R., Lasky J.R., Wagner H.H., Urban D.L. (2018). Comparing methods for detecting multilocus adaptation with multivariate genotype-environment associations. Mol. Ecol..

[24] Huang D.-F., He J.-W., Jiang Y.-S., Fu W.-T., Fan G.-L., Wu D., Zhan Y.-F., Shi Y.-J., Wang N.-Y. (2022). Analysis of SNP molecular markers in the whole genome of Niuchang pepper. Seed.

[25] Song J., Zhao X., Lin B., Zhang S., Lai H., Chen F., Huang D., Liu J., Hu H., Wang J. (2024). Single nucleotide polymorphisms and insertion/deletion variation analysis of octoploid and decaploid tropical oil tea *Camellia* populations based on whole-genome resequencing. Plants.

[26] Robinson K.M., Schiffthaler B., Liu H., Rydman S.M., Rendón-Anaya M., Kalman T.A., Kumar V., Canovi C., Bernhardsson C., Delhomme N. (2024). An improved chromosome-scale genome assembly and population genetics resource for *Populus tremula*. Physiol. Plant..

[27] Yocca A.E., Lu Z.-F., Schmitz R.J., Freeling M., Edger P.P. (2021). Evolution of conserved noncoding sequences in *Arabidopsis thaliana*. Mol. Biol. Evol..

[28] Jiao N.-B., Xu J.-R., Wang Y., Li D.-X., Chen F.-F., Chen Y., Chen J.-H. (2024). Genome-wide characterization of post-transcriptional processes related to wood formation in *Dalbergia odorifera*. BMC Genom..

[29] Pan Y., Luo F., Lu C.-H. (2014). Secondary substances and their ecological effects on seed dispersal in vertebrate dispersed fleshy fruit plants. Acta Ecol. Sin..

[30] Lin M., Liu X., Chen J., Huang J., Zhou L. (2024). Insecticidal triterpenes in Meliaceae III: Plant species, molecules, and activities in *Munronia*–*Xylocarpus*. Int. J. Mol. Sci..

[31] Lu C.-H. (2005). Chemical defense of fruits (seeds) and the adaptive strategies of frugivorous animals. Chin. J. Ecol..

[32] Dalling W.J., Davis S.A., Arnold E.A., Sarmiento C., Zalamea C.P. (2020). Extending plant defense theory to seeds. Annu. Rev. Ecol. Evol. Syst..

[33] Wang G.-H., Wei L.-J., Ya C.-Z., Zhong Y.-D., Tang C.-B. (2023). Feeding and dispersal of *Pycnonotus jocosus* on plant fruits in urban green space. J. Anhui Agric. Sci..

[34] Qiu Y.-X., Fu C.-X., Comes H.P. (2011). Plant molecular phylogeography in China and adjacent regions: Tracing the genetic imprints of Quaternary climate and environmental change in the world’s most diverse temperate flora. Mol. Phylogenet. Evol..

[35] Tollefsrud M.M., Myking T., Sønstebø J.H., Lygis V., Hietala A.M., Heuertz M. (2016). Genetic structure in the northern range margins of common ash, *Fraxinus excelsior* L.. PLoS ONE.

[36] Chen T.-Y., Lou A.-R. (2019). Phylogeography and paleodistribution models of a widespread birch (*Betula platyphylla* Suk.) across East Asia: Multiple refugia, multidirectional expansion, and heterogeneous genetic pattern. Ecol. Evol..

[37] Tollefsrud M.M., Kissling R., Gugerli F., Johnsen Ø., Skrøppa T., Cheddadi R., Van der Knaap W.O., Latałowa M., Terhürne-Berson R., Litt T. (2008). Genetic consequences of glacial survival and postglacial colonization in Norway spruce: Combined analysis of mitochondrial DNA and fossil pollen. Mol. Ecol..

[38] Edwards E.J., Chatelet D.S., Chen B.-C., Ong J.Y., Tagane S., Kanemitsu H., Tagawa K., Teramoto K., Park B., Chung K.-F. (2017). Convergence, consilience, and the evolution of temperate deciduous forests. Am. Nat..

[39] Wu Y., Yang K., Wen X., Sun Y. (2024). Genetic differentiation and relationship among *Castanopsis chinensis*, *C. qiongbeiensis*, and *C. glabrifolia* (Fagaceae) as revealed by nuclear SSR markers. Plants.

[40] Slatkin M. (1985). Gene flow in natural populations. Annu. Rev. Ecol. Syst..

[41] Hernández-Hernández T., Miller E.C., Román-Palacios C., Wiens J.J. (2021). Speciation across the tree of life. Biol. Rev..

[42] Pinheiro F., Veiga G.S., Chaves C.J.N., da Costa C.T., da Silva C.P. (2021). Reproductive barriers and genetic differentiation between continental and island populations of *Epidendrum fulgens* (Orchidaceae). Plant Syst. Evol..

[43] Ho K.-Y., Ko N.-Y. (2010). Genetic diversity among *Melia azedarach* L. populations at different climatic regions in Taiwan. Crop Environ. Bioinform..

[44] Feng L., Tian X.-N., El Kassaby Y.A., Qiu J., Feng Z., Sun J.-J., Wang G.-B., Wang T.-L. (2022). Predicting suitable habitats of *Melia azedarach* L. in China using data mining. Sci. Rep..

[45] Zhang Q.-J., Baiakhmetov E., Dai P.-G., Zhang Y.-Y., Wu X.-Y., Jiang X., Li Z.-H. (2026). Multi-omics of the endangered *Acer miaotaiense* reveal nervonic acid biosynthesis, population evolution and environmental adaptation. Plant Physiol..

[46] Soltabayeva A., Dauletova N., Serik S., Sandybek M., Omondi J.O., Kurmanbayeva A., Srivastava S. (2022). Receptor-like kinases (LRR-RLKs) in response of plants to biotic and abiotic stresses. Plants.

[47] Seo Y.-S., Lee S.-K., Song M.-Y., Suh J.-P., Hahn T.-R., Ronald P., Jeon J.-S. (2008). The HSP90–SGT1–RAR1 molecular chaperone complex: A core modulator in plant immunity. J. Plant Biol..

[48] de Villemereuil P., Gaggiotti O.E., Mouterde M., Till-Bottraud I. (2016). Common garden experiments in the genomic era: New perspectives and opportunities. Heredity.

